# Early Prediction of Massive Transfusion for Patients With Traumatic Hemorrhage: Development of a Multivariable Machine Learning Model

**DOI:** 10.1097/AS9.0000000000000314

**Published:** 2023-08-16

**Authors:** Andrew J. Benjamin, Andrew J. Young, John B. Holcomb, Erin E. Fox, Charles E. Wade, Chris Meador, Jeremy W. Cannon

**Affiliations:** From the * Division of Traumatology, Surgical Critical Care & Emergency Surgery, Department of Surgery, Perelman School of Medicine at the University of Pennsylvania, Philadelphia, PA; † Trauma and Acute Care Surgery, Department of Surgery, The University of Chicago, Chicago, IL (Current affiliation); ‡ Division of Trauma, Critical Care and Burn, Department of Surgery, The Ohio State University Wexner Medical Center, Columbus, OH; § Division of Trauma and Acute Care Surgery, Department of Surgery, University of Alabama at Birmingham, Birmingham, AL; ‖ Department of Surgery, F. Edward Hébert School of Medicine at the Uniformed Services University, Bethesda, MD; ¶ Center for Translational Injury Research and Division of Acute Care Surgery, Department of Surgery, McGovern Medical School, University of Texas Health Science Center at Houston, Houston, TX; # Arcos, Inc., Missouri City, TX; ** Leonard Davis Institute of Health Economics, University of Pennsylvania, Philadelphia, PA.

## Abstract

**Objective:**

Develop a novel machine learning (ML) model to rapidly identify trauma patients with severe hemorrhage at risk of early mortality.

**Background:**

The critical administration threshold (CAT, 3 or more units of red blood cells in a 60-minute period) indicates severe hemorrhage and predicts mortality, whereas early identification of such patients improves survival.

**Methods:**

Patients from the PRospective, Observational, Multicenter, Major Trauma Transfusion and Pragmatic, Randomized Optimal Platelet, and Plasma Ratio studies were identified as either CAT+ or CAT−. Candidate variables were separated into 4 tiers based on the anticipated time of availability during the patient’s assessment. ML models were created with the stepwise addition of variables and compared with the baseline performance of the assessment of blood consumption (ABC) score for CAT+ prediction using a cross-validated training set and a hold-out validation test set.

**Results:**

Of 1245 PRospective, Observational, Multicenter, Major Trauma Transfusion and 680 Pragmatic, Randomized Optimal Platelet and Plasma Ratio study patients, 1312 were included in this analysis, including 862 CAT+ and 450 CAT−. A CatBoost gradient-boosted decision tree model performed best. Using only variables available prehospital or on initial assessment (Tier 1), the ML model performed superior to the ABC score in predicting CAT+ patients [area under the receiver-operator curve (AUC = 0.71 vs 0.62)]. Model discrimination increased with the addition of Tier 2 (AUC = 0.75), Tier 3 (AUC = 0.77), and Tier 4 (AUC = 0.81) variables.

**Conclusions:**

A dynamic ML model reliably identified CAT+ trauma patients with data available within minutes of trauma center arrival, and the quality of the prediction improved as more patient-level data became available. Such an approach can optimize the accuracy and timeliness of massive transfusion protocol activation.

## Abstract 2

Mini-Abstract

We developed a novel machine learning model to identify patients with severe bleeding within minutes of trauma center arrival. In patients included in the PRospective, Observational, Multicenter, Major Trauma Transfusion and Pragmatic, Randomized Optimal Platelet, and Plasma Ratios studies, a CatBoost gradient-boosted decision tree model demonstrated superior performance compared with the assessment of blood consumption score (area under the receiver-operator curve = 0.81 vs 0.62). Discrimination ability improved as more patient-level data became available.

## INTRODUCTION

Hemorrhagic shock results in nearly 50,000 deaths annually in the United States and nearly 1.5 million worldwide.^1^ Although advances in resuscitation have improved survival, mortality remains near 25%, even in young otherwise healthy patients^2,3^ leading some to question the true benefit of these recent advances.^4^ Timely recognition of hemorrhagic shock and initiation of blood product transfusion serve to improve survival when combined with surgical hemostasis.^5^ Blood product refrigerators and massive transfusion protocols (MTP) facilitate early blood product delivery to the bedside of acutely bleeding patients, and these measures have become the standard of care at trauma centers.^6^

Nonetheless, activating the MTP in the correct patient remains challenging.^7^ On the one hand, over-activation of MTPs costs the blood bank time and effort that could be spent performing other clinical support activities and leads to increased blood product wastage, which is particularly problematic amid the current nationwide blood product shortage. Conversely, delaying MTP activation for a patient with a life-threatening occult hemorrhage can delay the delivery of blood products for resuscitation, which is associated with increased mortality.^5^ Thus, early identification of hemorrhagic shock and the need for massive transfusion (MT) is critical to patient survival.^8–10^ Clinical gestalt alone fails to correctly identify up to 30% of patients who ultimately require an MT whereas over-activating MTP for many others.^11^ Therefore, a number of scoring systems have been developed as clinical decision-support aids to help predict the need for massive transfusion.^7,12^ Although these scores are often used in research studies, many have significant limitations and fail to maximize sensitivity to avoid missing patients with occult bleeding balanced against the specificity needed to limit resource overutilization in everyday clinical practice.

Machine learning (ML), a subfield of artificial intelligence, facilitates the rapid synthesis of highly granular data to make predictions in real-time by utilizing associations that are not readily apparent or easily calculated by the trauma team at the bedside. Furthermore, ML algorithms can improve over time and adapt to local center-level nuances as more data is collected and used to update the algorithm. Due to the high mortality rate of hemorrhagic shock and the short time to death in patients with severe hemorrhage,^13^ even small improvements in accurately identifying those with occult hemorrhage can have large benefits, while also limiting false alarms that waste limited resources. In this study, we hypothesized that an ML algorithm that predicts MT events could be developed to successfully identify patients who suffer early death from hemorrhage or who require large volume blood product resuscitation with greater accuracy and precision than conventional MT prediction scores.

## MATERIALS AND METHODS

### Study Population

This study was conducted in accordance with the Transparent Reporting of a multivariable prediction model for Individual Prognosis or Diagnosis guidelines (Supplemental Table, http://links.lww.com/AOSO/A523). Data from 2 studies with detailed blood product administration and patient physiology records were used to build the dynamic blood navigator ML model (BloodNav-MLM): the PRospective, Observational, Multicenter, Major Trauma Transfusion (PROMMTT) study and the Pragmatic, Randomized Optimal Platelet and Plasma Ratios (PROPPR) trial, both of which are described in detail elsewhere.^2,3^ In the present study, patients from the PROMMTT and PROPPR were included if they had a focused assessment with sonography for trauma (FAST) examination performed and received at least 1 unit of blood.

Briefly, the PROMMT study was a prospective observational study conducted at 10 US Level 1 trauma centers between July 2009 and October 2010. All participating centers had preexisting MTPs in place but demonstrated significant variations in transfusion practice. The study subjects were at least 16 years of age, required the highest level of trauma activation, and received at least 1 unit of packed red blood cells (PRBCs) within the first 6 hours of admission. Patients were excluded if they were transferred from another facility, pronounced dead within 30-minutes of arrival, had more than 5 minutes of cardiopulmonary resuscitation before or within the first 30-minutes of admission, had burn injuries over 20% of the total body surface area, presented with an inhalation injury, were prisoners, or were pregnant. Of the 12,560 patients screened, 1245 were enrolled in the study.

The PROPPR study was a multicenter randomized clinical trial conducted at 12 North American level 1 trauma centers from August 2012 to December 2013. Patients were randomized at each site into 2 groups to receive blood products at a ratio of 1:1:1 or 1:1:2. In the 1:1:1 group, patients first received pooled 6 units of platelets, followed by alternating PRBCs and plasma up to 6 times. For the 1:1:2 group, patients received alternating 2 units of PRBCs with 1 unit of plasma up to 3 times, a pooled 6 units of platelets, followed by alternating 2 units of PRBCs with 1 unit of plasma an additional 3 times. Patients were transfused until hemostasis was obtained, death occurred, or there was no need for further transfusion. Patients were followed up until discharge or up to 30 days of hospitalization. Of the 11,185 patients screened, 680 were randomized (1:1:1, n = 338; 1:1:2, n = 342) and included in this study.

### Definitions

Historically, MT was defined as 10 or more units of whole blood within 24 hours.^14,15^ With the advent of component blood products, it was modified to 10 or more units of PRBCs in a 24-hour period.^16,17^ Although this definition is commonly used clinically and in research, it suffers from survival bias (patients may expire before they have time to receive 10 units of PRBCs) and fails to identify patients with severe hemorrhage who achieve early hemostasis with rapid surgical intervention and balanced blood product resuscitation. An alternative MT definition is the critical administration threshold (CAT), defined as 3 or more units of PRBCs in a 1-hour period.^18^ This definition is a more sensitive indicator of MT as well as a better predictor of mortality from acute hemorrhage than traditional definitions of MT. Because it is a prospective continuous measurement, CAT better reflects the severity of hemorrhage and the intensity of blood product resuscitation while minimizing survivor bias.

Consequently, for this analysis, we used CAT positivity (CAT+) within 24 hours of presentation as the primary endpoint.^19^ Secondary endpoints included multiple CAT events (defined as 2 or more CAT+ events within 24 hours but not spanning the same hour), receipt of a traditional MT (≥10 units PRBC in 24 hours), and 6-hour mortality.

### Machine Learning Model and Statistical Methods

Model predictions require an assessment of the need for MTP activation within minutes of emergency department (ED) arrival. To build our BloodNav-MLM, we stratified the candidate model inputs into 4 tiers based on the timeliness of their availability (Table 1). Tier 1 variables were defined as those available upon presentation or after the primary survey. Tier 2 variables were defined as the vital signs and adjuncts to the primary survey. Tier 3 variables were defined as lab values available within 5 minutes or point-of-care results. Finally, tier 4 variables were defined as all remaining laboratory data collected during the initial patient assessment in the ED.

**TABLE 1.** Model Input Definitions and Model Variables

	Tier 1	Tier 2	Tier 3	Tier 4
Definition	Available prehospital/on presentation	Additional vital signs and adjuncts	Laboratory values available within 5 minutes	Laboratory values available after 5 minutes
Variables	Age Sex Prehospital crystalloids Systolic blood pressure Diastolic blood pressure Heart rate GCS Penetrating mechanism	Temperature Respiratory rate Positive FAST Chest bleeding	pH Base deficit Lactate	Hemoglobin PTT INR Na^+^K^+^Cl^-^BUN Cr Glucose

BUN indicates blood urea nitrogen; FAST, Focused Assessment with Sonography in Trauma; GCS, Glasgow Coma Scale; INR, international normalized ratio; PTT, partial thromboplastin time.

Categorical variables were compared using *χ*^2^ tests, and continuous variables were evaluated using the Wilcoxon rank sum test as all variables were non-normally distributed by the Shapiro-Wilk test, with a *P* value less than 0.05, denoting statistical significance. All univariate analyses were performed using R 4.0.3 (R Foundation for Statistical Computing, Vienna, Austria). We used a tree-based pipeline optimization tool,^20^ an automated model selection platform built on scikit-learn^21^ in Python 3.6, to test multiple ML methods for predicting CAT+ patients. The models tested included logistic regression, gradient-boosted decision trees, random forest, and k-nearest neighbor. To measure the utility of using probability predictions to make binary yes/no decisions, we measured the area under the receiver-operator curve (AUC) and the F1 score (a measure of the harmonic mean of precision and recall).

The data were randomly split into training (80%) and test (20%) sets for model development, stratifying the positive outcome to keep the rate equal between the splits. Models were then constructed by stepwise addition of variables from each tier of variables and tuned using Optuna, an automated hyperparameter optimization framework.^22^ Class weights were adjusted in the training process to account for the degree of class imbalance. The performance of each candidate ML model was compared with the performance of the assessment of blood consumption (ABC) score using a threshold score of ≥2.^12^ The ABC score is a validated tool to predict the need for MT in trauma patients. It is based on 4 criteria: penetrating mechanism of injury, systolic blood pressure (SBP) ≤90 mmHg, heart rate ≥120 beats per minute, and positive FAST examination. Each criterion is assigned 1 point, and the total score ranges from 0 to 4. A score of ≥2 indicates a high risk of MT and triggers the activation of an MTP. The AUC with a 95% confidence interval (CI) was calculated for predictions of CAT+ using bootstrapping methods with 1000 iterations. *P* values were calculated to compare AUCs for each ML model with the ABC score using 2-tailed t-tests. A *P* value less than 0.05 was considered statistically significant. Other performance metrics were calculated based on thresholds that maximized the Matthews Correlation Coefficient.^23^ The calibration of the final model was assessed by plotting the predicted probabilities derived from the model versus the actual probabilities. The relative importance of features in the model was assessed by calculating Shapley additive explanation feature importance scores on Tier 1 variables and the full model.

## RESULTS

### Patient Characteristics

**FIGURE 1.**
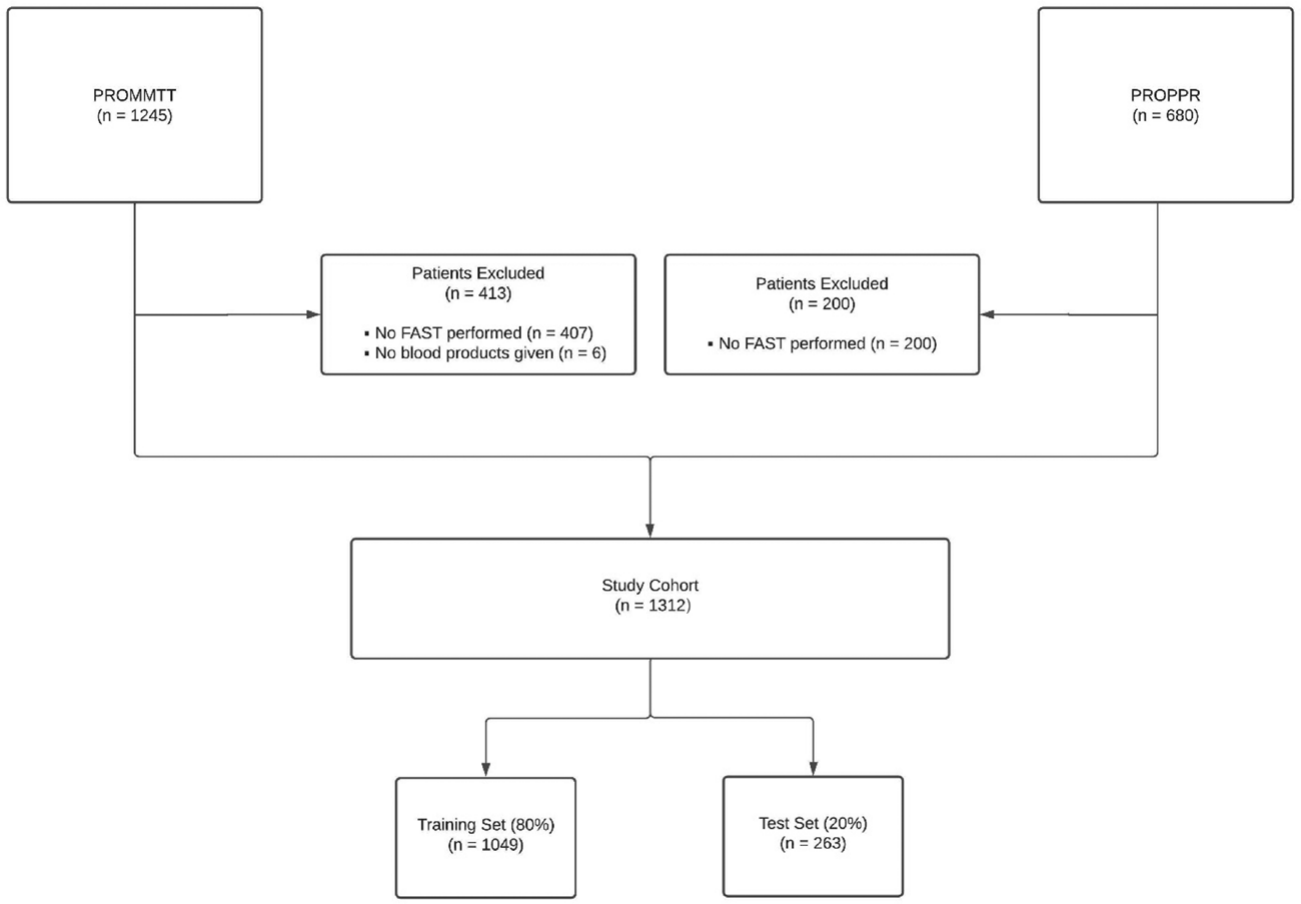
Study flow diagram.

The combined dataset contained 1312 patients including 862 CAT+ patients and 450 CAT− patients (**Fig. 1**). Of the variables included in the univariate analysis, the 2 cohorts were significantly different in all categories except for age, temperature, respiratory rate, chest bleeding, serum potassium, and blood urea nitrogen (**Table 2**) although the distribution of the model input variables demonstrated only subtle differences between CAT+ and CAT− patients (Supplemental Figure 1, http://links.lww.com/AOSO/A229). The data were then randomly divided into a training set (N = 1049) for model development and a hold-out test set (N = 263), with patients stratified by CAT status.

**TABLE 2.** Cohort Demographics, Presenting Model Variable Values and Outcomes

	**N**	**Overall (N = 1312**)	**CAT+ (N = 862**)	**CAT− (N = 450**)	***P* value**
Age	1312	37 (25, 53)	37 (24, 52)	38 (25, 54)	0.098
Male, n (%)	1312	981/1312 (75%)	665/862 (77%)	316/450 (70%)	0.006
Prehospital crystalloids (mL)	1312	400 (41.75, 1,000)	400 (0, 1,000)	500 (100, 1,100)	0.004
SBP	1294	104 (83, 127)	100 (80, 124)	110 (90, 135)	<0.001
DBP	1091	68 (51, 85)	65 (50, 87)	70 (58, 83)	0.039
HR	1304	108 (89, 128)	112 (91, 131)	102 (83, 123)	<0.001
GCS	1253	13 (3, 15)	12 (3, 15)	14 (3, 15)	0.003
Penetrating mechanism, n (%)	1312	388 (30%)	271 (31%)	117 (26%)	0.040
Temperature (°C)	639	36.2 (35.60, 36.6)	36.2 (35.6, 36.6)	36.3 (35.6, 36.6)	0.451
RR	909	21 (18, 26)	22 (18, 27)	21 (18, 26)	0.235
Positive FAST, n (%)	1312	462 (35%)	359 (42%)	103 (23%)	<0.001
Chest bleeding, n (%)	1312	335 (26%)	228 (26%)	107 (24%)	0.292
pH	1125	7.25 (7.15, 7.33)	7.22 (7.12, 7.30)	7.29 (7.23, 7.36)	<0.001
Base deficit	1114	−7 (−11 to −3)	−8 (−12 to −4)	−5 (−8 to −2)	<0.001
Lactate	621	5.0 (3.2, 7.7)	5.5 (3.5, 8.6)	3.6 (2.7, 5.6)	<0.001
Hgb	1282	12.0 (10.3, 13.5)	11.7 (10.1, 13.2)	12.4 (10.9, 14.0)	<0.001
PTT	1007	28 (25, 34)	30 (26, 36)	27 (24, 30)	<0.001
INR	1055	1.2 (1.1, 1.5)	1.3 (1.2, 1.6)	1.2 (1.1, 1.3)	<0.001
Na^+^	1267	140 (138, 142)	141 (138, 143)	140 (137, 142)	<0.001
K^+^	1262	3.7 (3.3, 4.1)	3.7 (3.3, 4.1)	3.7 (3.3, 4.0)	0.563
Cl^-^	1199	106 (104, 109)	107 (104, 109)	106 (103, 109)	0.003
BUN	1227	14 (11, 17)	14 (11, 17)	14 (11, 17)	0.893
Cr	1234	1.2 (1.0, 1.4)	1.2 (1.0, 1.5)	1.1 (0.9, 1.3)	<0.001
Glucose	1274	175 (142, 226)	188 (150, 239)	157 (132, 201)	<0.001
ISS	1312	27 (17, 38)	29 (19, 41)	24.50 (14, 34)	<0.001
6-hr PRBC	1310	5 (2, 9)	8 (5, 13)	2 (1, 3)	<0.001
6-hr FFP	1284	3 (1, 7)	5 (3, 10)	0 (0, 2)	<0.001
6-hr PLT	1207	0 (0, 6)	6 (0, 12)	0 (0, 0)	<0.001
24-hr PRBC	1312	6 (3, 11.25)	9 (6, 16)	2 (2, 4)	<0.001
24-hr FFP	1312	4 (2, 9)	7 (3, 13)	2 (0, 4)	<0.001
24-hr PLT	1312	1 (0, 7.50)	6 (0, 12)	0 (0, 0)	<0.001
MT+, n (%)	1312	426 (32%)	412 (48%)	14 (3.1%)	<0.001
6-hr morality, n (%)	1312	54 (4.1%)	52 (6.0%)	2 (0.4%)	<0.001

Median (IQR) or n (%).

BUN indicates blood urea nitrogen; CAT, critical administration threshold; DPB, diastolic blood pressure; FAST, Focused Assessment with Sonography in Trauma; FFP, fresh frozen plasma; GCS, Glasgow Coma Scale; Hgb, hemoglobin; HR, heart rate; INR, international normalized ratio; ISS, Injury Severity Score; MT+, massive transfusion by traditional definition of 10 units PRBC in 24 hours; PRBC, packed red blood cells; PLT, platelets; PTT, partial thromboplastin time; RR, respiratory rate; SBP, systolic blood pressure.

### ML Prediction Performance

**FIGURE 2.**
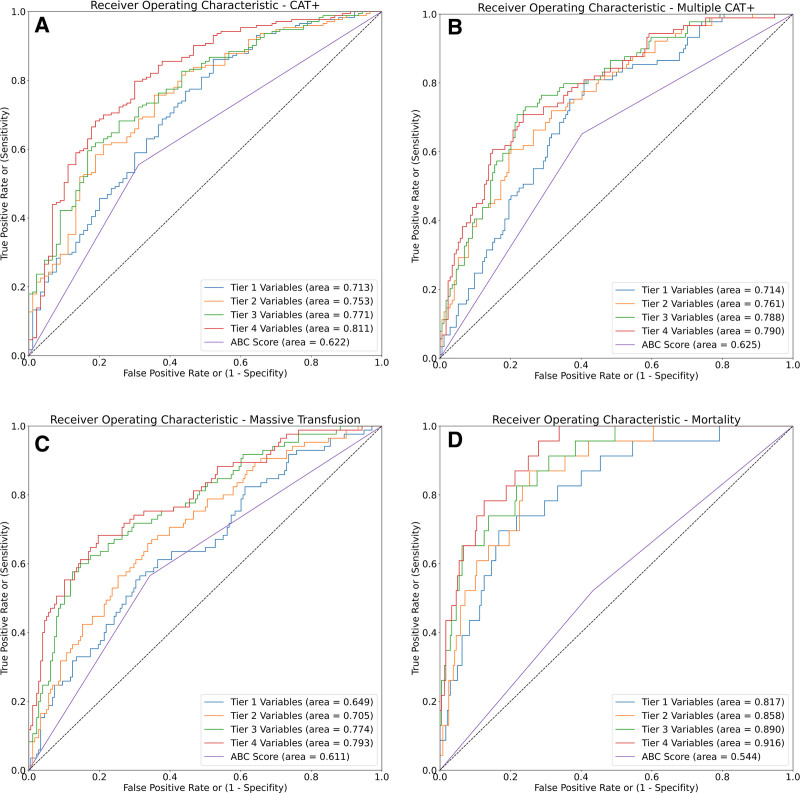
Receiver-operator curves for predicting (A) CAT+, (B) multiple CAT+, (C) massive transfusion, and (D) 6-hour mortality. CAT indicates critical administration threshold.

The candidate variables included in the model were separated into tiers, as described above (**Table 1**). Automated model selection with tree-based pipeline optimization tool identified CatBoost, a gradient-boosted decision tree model that inherently handles missingness, to perform the best in predicting CAT+ patients (Supplemental Data, http://links.lww.com/AOSO/A229).^24^ Using Tier 1 variables, the model outperformed the ABC score in predicting CAT+ patients, with an AUC of 0.71 (95% CI = 0.65–0.78) compared to 0.62 (95% CI = 0.56–0.68*; P* = 0.02). With the addition of each tier of variables, the discrimination of the model improved, further outperforming the ABC score with an AUC of 0.75 for Tier 2 (95% CI = 0.69–0.82; *P* < 0.001), 0.77 for Tier 3 (95% CI = 0.71–0.83*; P* < 0.001), and 0.81 for Tier 4 (95% CI = 0.75–0.87; *P* < 0.001) (**Fig. 2**). A comprehensive list of the accuracy, precision, recall, F1 score, and positive and negative predictive values is shown for each tier of the variables in **Table 3**.

**TABLE 3.** Comparison of Machine Learning Model Predictions

Model	ABC	ML Tier 1	ML Tier 2	ML Tier 3	ML Tier 4
A) Model performance for CAT					
Accuracy	0.60	0.73	0.73	0.74	0.78
Sensitivity	0.55	0.93	0.94	0.87	0.94
Specificity	0.68	0.36	0.33	0.57	0.46
PPV	0.77	0.73	0.73	0.77	0.77
NPV	0.45	0.74	0.74	0.66	0.80
F1 Score	0.64	0.82	0.82	0.81	0.85
AUC	0.62	0.71	0.75	0.77	0.81
B) Model performance for multiple CAT+ events					
Accuracy	0.62	0.65	0.73	0.74	0.77
Sensitivity	0.65	0.81	0.61	0.71	0.71
Specificity	0.60	0.56	0.80	0.76	0.78
PPV	0.40	0.49	0.61	0.60	0.62
NPV	0.78	0.85	0.80	0.84	0.84
F1 Score	0.53	0.61	0.61	0.65	0.66
AUC	0.63	0.71	0.76	0.79	0.79
C) Model performance for massive transfusion					
Accuracy	0.63	0.63	0.66	0.77	0.76
Sensitivity	0.66	0.61	0.66	0.60	0.68
Specificity	0.44	0.67	0.66	0.85	0.80
PPV	0.44	0.44	0.48	0.66	0.62
NPV	0.76	0.77	0.80	0.82	0.84
F1 Score	0.49	0.51	0.56	0.63	0.65
AUC	0.61	0.65	0.71	0.77	0.79
D) Model performance for 6-hour mortality					
Accuracy	0.49	0.82	0.89	0.91	0.92
Sensitivity	0.62	0.70	0.52	0.65	0.61
Specificity	0.57	0.81	0.77	0.94	0.95
PPV	0.06	0.28	0.41	0.50	0.52
NPV	96	0.97	0.95	0.97	0.96
F1 Score	0.11	0.40	0.46	0.56	0.56
AUC	0.55	0.82	0.86	0.89	0.92

AUC indicates area under the receiver-operator curve; CAT, critical administration threshold; NPV, negative predictive value; PPV, positive predictive value.

For predicting multiple CAT+ events, BloodNav-MLM outperformed the ABC score when using Tier 1 variables only, with an AUC of 0.71 (95% CI = 0.65–0.78) compared with 0.62 (95% CI = 0.56–0.67; *P* = 0.01). Again, the discrimination of the model improved with each tier of variables, with the Tier 4 model having an AUC of 0.79 (95% CI = 0.73–0.84) (**Fig. 2**) and an F1 score of 0.66 (**Table 3**). For predicting traditional massive transfusion, the BloodNav-MLM Tier 1 model performed similarly to the ABC score, with an AUC of 0.65 (95% CI = 0.58–0.72) compared with 0.61 (95% CI = 0.55–0.67; *P* = 0.34). The addition of Tier 2, Tier 3, and Tier 4 variables improved the performance of the model, with AUCs of 0.71 (95% CI = 0.64–0.77; *P* = 0.01), 0.77 (95% CI = 0.71–0.83; *P* < 0.001) and 0.79 (95% CI = 0.73–0.85; *P* < 0.001), respectively (**Table 3**).

Finally, for predicting 6-hour mortality, BloodNav-MLM outperformed the ABC score using Tier 1 variables with an AUC of 0.82 (95% CI = 0.73–0.90) compared to 0.55 (95% CI = 0.50–0.66*; P* < 0.001). In addition, the BloodNav-MLM has a significantly better F1 score (**Table 3**). The addition of Tiers 2, 3, and 4 improved the performance of the model, with the final Tier 4 model having an AUC of 0.92 (95% CI = 0.87–0.96; *P* < 0.001) and an F1 score of 0.56 (**Table 3**).

**FIGURE 3.**
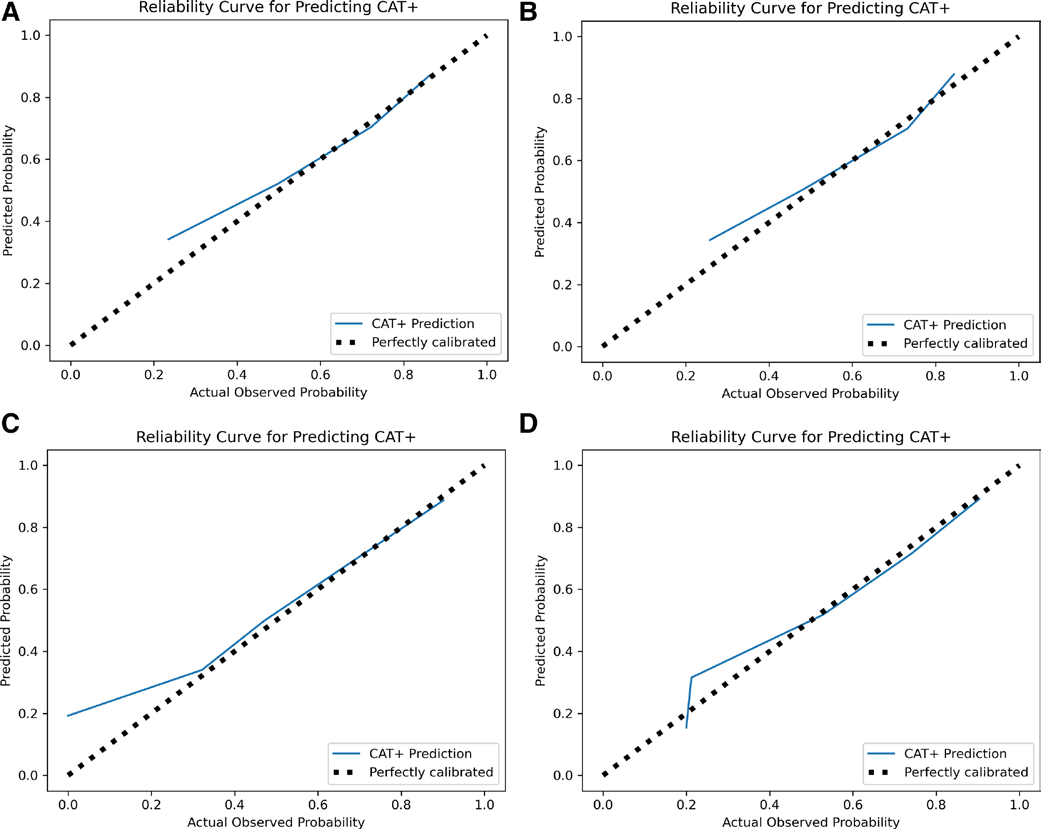
Calibration curves for CAT+ prediction by variable tier: (A) Tier 1, (B) Tier 2, (C) Tier 3, and (D) Tier 4. CAT indicates critical administration threshold.

**FIGURE 4.**
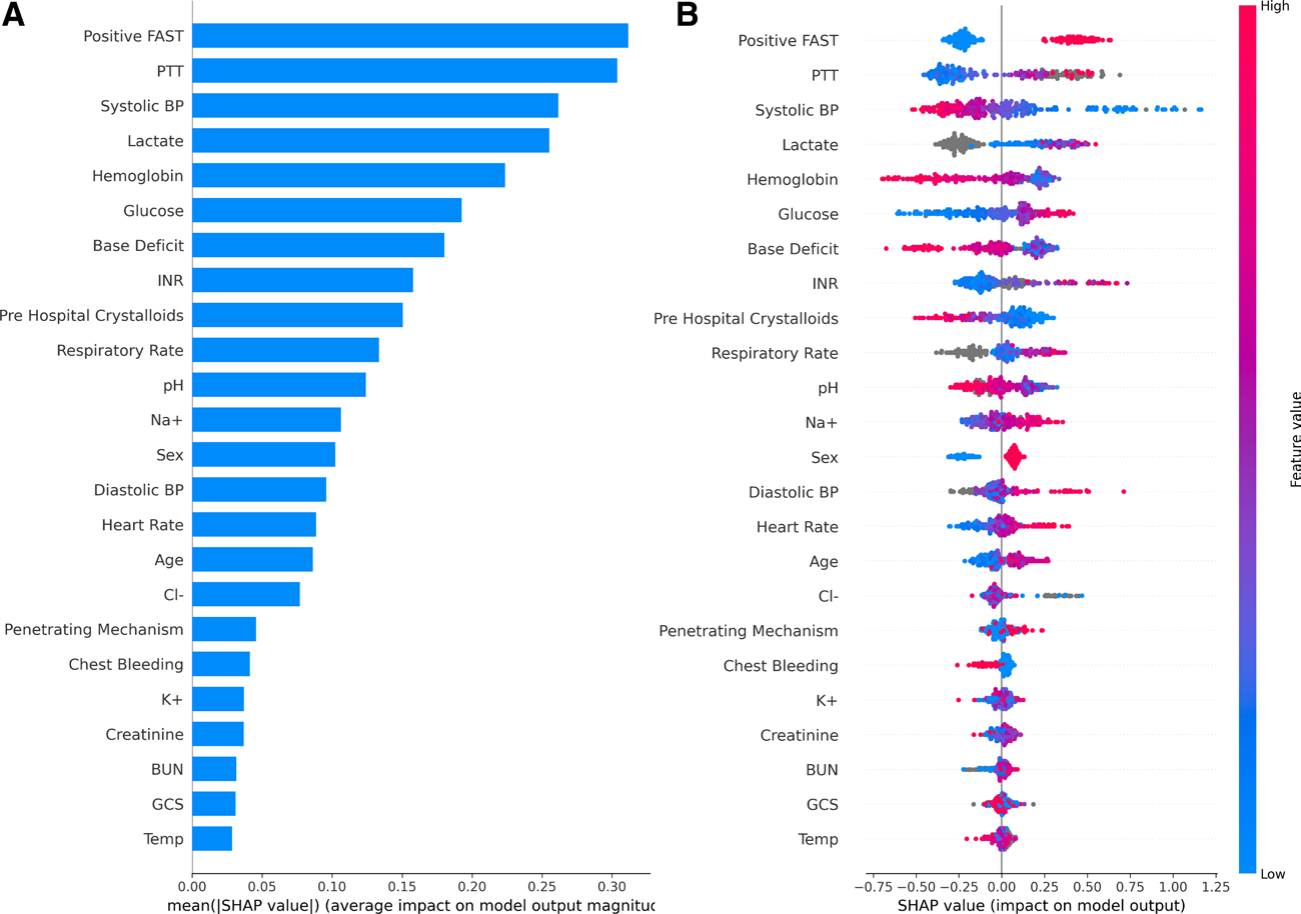
Feature importance assessment for the model variables using Shapley additive explanation (SHAP) methods. A, Contribution of each variable to the model’s decision-making ranked by SHAP value. B, Beeswarm plots demonstrating the association of each variable with the prediction of a CAT+ event, as identified by the model. Each point on the SHAP plot corresponds to an individual patient. The collective of points on the SHAP plot creates a beeswarm that represents how the model associates each variable with a CAT+ event based upon the value of the variable.

**Figure 3** shows the calibration curves for each model’s performance in the validation cohort for predicting CAT+ events. The models provided excellent predictions throughout the full range of CAT+ risk. The Tier 1 model slightly over-predicted CAT+ events for probabilities over 50%, and Tier 3 slightly over-predicted for probabilities under 35%. Shapley additive explanation feature importance analysis demonstrated that for the Tier 1 model, prehospital crystalloids, SBP, heart rate, and diastolic blood pressure were the most important features (Supplemental Figure 2, http://links.lww.com/AOSO/A229) whereas SBP, positive FAST, lactate, and partial thromboplastin time were the most important inputs in the final model (**Fig. 4**).

Model performance was assessed across the entire range of CAT+ probability thresholds using the 20% validation set (Supplemental Figure 3, http://links.lww.com/AOSO/A229). Model accuracy was maximized at a CAT+ probability threshold of 42% at which the model misclassified 10 patients as CAT− who were CAT+ (5.8% false negative) and 49 patients as CAT+ who were CAT− (54.4% false positive).

## DISCUSSION

Hemorrhagic shock remains the leading cause of mortality within the first 6–24 h after a traumatic injury. MTP provides a survival benefit for patients in hemorrhagic shock,^6^ but must be activated promptly to confer any mortality benefit.^5^ Yet identifying patients at risk for massive hemorrhage remains extremely challenging. In this secondary analysis of the PROMMTT study and PROPPR trial, we developed the BloodNav-MLM, the first ML model to leverage subtle differences between CAT+ and CAT− patients to accurately identify those with even occult hemorrhage within minutes of patient arrival to the trauma bay. The quality of the prediction is dynamic, with the ability to improve in real-time as additional data becomes available. Such an approach may aid in improving the timeliness and accuracy of massive transfusion protocol activation, augmented by clinician judgment. More importantly, compared to simple scoring systems that bin or discretize variables to allow score calculation at the bedside, an ML approach can instantly synthesize complex, granular data to provide highly accurate predictions. Our algorithm demonstrated excellent discriminatory ability, even when using simple Tier 1 variables available immediately upon presentation. In fact, all Tier 1 variables could be input into the model before the patient’s arrival to the ED based on prehospital data, thereby guiding prehospital resuscitation and early activation of the receiving trauma center’s MTP. The inclusion of additional data available as the resuscitation unfolds—including several novel inputs not intuitively linked to acute hemorrhage—further improved the predictive accuracy of this algorithm.

Numerous investigators have developed rubrics to predict the need for MT upon presentation to the trauma bay. Examples include the Trauma-Associated Severe Hemorrhage score, the Revised Assessment of Bleeding and Transfusion score, and the widely used ABC score.^12,25^ The ABC score benefits from simplicity, with its scoring parameters being easily calculated at the bedside. However, this score was created and validated using an MT definition of ≥10 units of PRBCs in a 24-hour period, as opposed to CAT, which is more useful as a marker of the need for MTP activation in real-time and significantly more predictive of mortality as compared to traditional definitions of MT.^18^ When evaluated in the PROMMTT study, the ABC score demonstrated suboptimal sensitivity and specificity.^11^ Fundamentally, scores such as the ABC score trade nuance for simplicity by converting continuous variables to discrete categorical values to increase ease of use. This tradeoff can facilitate early MTP initiation but comes at the expense of accuracy. Poor predictive accuracy can overestimate the need for blood products, thereby potentially increasing product waste compared with physician judgment or alternative scoring systems.^26,27^ Setting an inappropriately high score threshold to trigger MTP activation can also lead to resuscitation delays and potentially increased mortality in patients who present with occult shock.

To address these limitations, Mina et al^28^ created an MT prediction model using continuous values for HR and SBP as opposed to discretized categorical versions of these values. This model also uses 3 mechanism types (blunt, stab, and gunshot) and adds a base deficit akin to our approach. Although the initial assessment of this single-center derived model indicated very favorable performance when predicting MTP activation (AUC = 0.96), external validation against multicenter data found significantly decreased performance when predicting MT by any of several definitions.^29^ Subsequent implementation of this prediction model further demonstrated relatively poor sensitivity and specificity, resulting in potential activation delays in 15 of 40 (37.5%) MT+ patients and over-activation in 28 of 281 (10%) MT− patients.^29^ In contrast, our model was based on data from 16 unique trauma centers across 2 studies and allows the use of additional inputs as they become clinically available, including examination elements (GCS and FAST results) and multiple laboratory values. This approach provided an AUC of up to 0.81 when predicting CAT+ events signifying early acute hemorrhage.

In practice, our BloodNav-MLM could be fully integrated into the electronic health record to automatically extract results from the patient’s chart and provide real-time feedback to trauma teams actively caring for critical patients. Automatic score generation would both decrease the error rate in score calculation and allow the score to be updated as new variables become available, thereby immediately alerting teams when the initiation of MTP would be prudent. Although the algorithm was constructed using variable tiers that accounted for the time of availability, it could be modified and continuously calculated to capture a subset of patients who will have a delayed need for MTP, extending its usefulness beyond the trauma bay. Our model can also be modified to incorporate measures of unique center-level characteristics.

The strengths of this study are the use of a cohort of patients from 2 multicenter studies that had rigorous selection criteria and the use of variables that were collected in a highly protocolized manner from many different trauma centers in the United States, thereby increasing the generalizability of our results. In addition, our model uses advanced predictive analytical techniques and contains variables that are commonly collected and used in standard practice. In addition, unlike prior studies that have predicted large volume traditional MT events subject to survivor bias, our model predicts CAT+ events, which represent an earlier and more accurate indicator of both severe and occult hemorrhage.^30^ However, there are several limitations to our study. Although the model development included both training and test sets, all the data used for training and validation originated from the same source with inherent biases and potential inaccuracies. One such bias is that all patients in this cohort received at least 1 unit of PRBCs, meaning low-risk patients were excluded from the analysis. Thus, it is important to perform further validation on an external set of data to prospectively evaluate this model as others have done.^29^ Next, although the calibration plot showed that the model performed remarkably well over a broad range of CAT+ probabilities, accuracy suffered some in the high range of the Tier 1 model and the low range of the Tier 2 model. In practice, the model could simply relay a range of risks on the higher end and then, on the low end, indicate a risk of “<35%” or “low” probability and remain clinically useful. Finally, CAT+ events ultimately represent an indirect surrogate for acute hemorrhage that relies upon the recognition of acute hemorrhage and subsequent treatment intervention. Nonetheless, this endpoint represents an improvement over other historic approaches, as discussed above.

In conclusion, we successfully developed a model that predicts CAT+ events using readily available clinical data in a tiered manner. The model has improved accuracy for identifying these events when compared to a validated model such as the ABC score, and once externally validated, it could be implemented within the electronic health records to initiate massive transfusion protocols more rapidly and accurately, potentially improving patient outcomes while minimizing blood product waste.

## ACKNOWLEDGMENTS

The authors gratefully acknowledge Jordan Wolf who performed some of the initial data analysis and provided invaluable guidance on early iterations of the methodology.

A.J.B., J.B.H., E.E.F., C.E.W., and J.W.C: participated in the performance of the research. A.J.B., J.W.C.: participated in the writing of the article: All authors. Participated in research design, data analysis (E.E.F. is an epidemiologist), and critical review/revision of the article.

## Supplementary Material


