# Supplementary material for: Early Prediction of Massive Transfusion for Patients With Traumatic Hemorrhage: Development of a Multivariable Machine Learning Model
Source: Ann Surg Open. 2023 Aug 16;4(3):e314. doi: 10.1097/AS9.0000000000000314 (PMC10513183; doi:10.1097/AS9.0000000000000314)
Supplement: Supplementary file 1 [file as9-4-e314-s001.pdf]

Machine Learning Improves Accuracy of Massive Transfusion Prediction for Patients with Traumatic Hemorrhagic Shock

Supplemental Content

Andrew J. Benjamin, MD[1,2]*
Andrew J. Young, MD[3]
John B. Holcomb, MD[4,5]
Erin E. Fox, PhD[6]
Charles E. Wade, PhD[6]
Chris Meador, MBA[7]
**Jeremy W. Cannon, MD, SM[1,5,8]

[1]Department of Surgery, Division of Traumatology, Surgical Critical Care & Emergency Surgery, Perelman School of Medicine at the University of Pennsylvania, Philadelphia, PA, USA

[2]Trauma and Acute Care Surgery, Department of Surgery, The University of Chicago, Chicago, IL, USA (*Current affiliation)

[3]Division of Trauma, Critical Care & Burn, Department of Surgery, The Ohio State University Wexner Medical Center, Columbus, OH, USA

[4]Division of Trauma and Acute Care Surgery, Department of Surgery, University of Alabama at Birmingham, Birmingham, AL, USA

[5]Department of Surgery, F. Edward Hébert School of Medicine at the Uniformed Services University, Bethesda, MD, USA

[6]Center for Translational Injury Research and Division of Acute Care Surgery, Department of Surgery, McGovern Medical School, University of Texas Health Science Center at Houston, Houston, TX, USA

[7]Arcos, Inc., Missouri City, TX, USA

[8]Leonard Davis Institute of Health Economics, University of Pennsylvania, Philadelphia, PA, USA

****Corresponding Author and Contact for Reprint Requests:**
Jeremy W. Cannon, MD, SM
51 N. 39th Street, Medical Office Building 120
Philadelphia, PA 19104
215-662-7320
FAX: 215-662-9471
jeremy.cannon@pennmedicine.upenn.ed

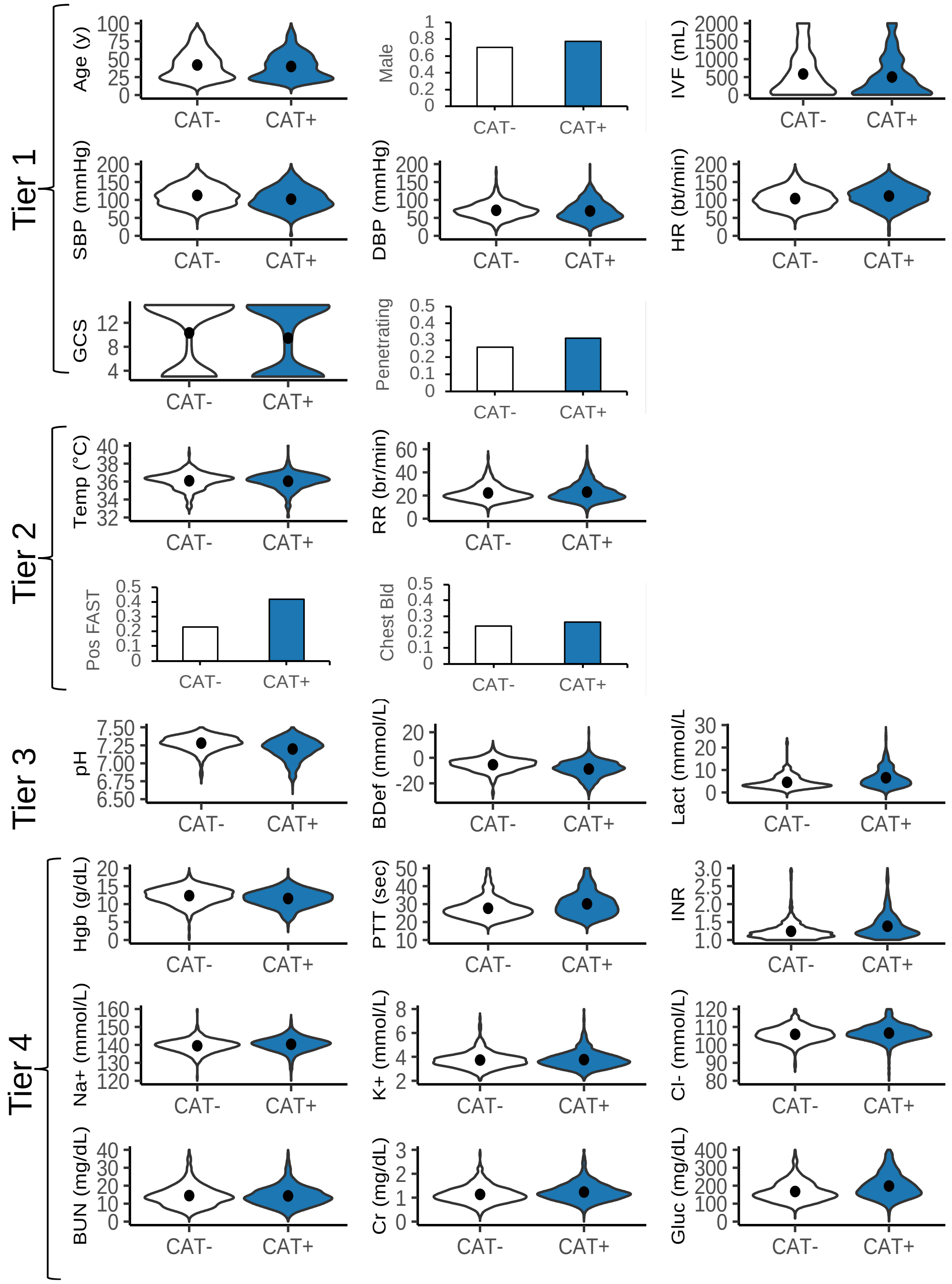

Supplemental Figure 1. Comparison of model variables between CAT- and CAT+ patients. Plots represent frequency distributions (kernel density plots) or relative frequency (bar plots) with patients sorted by CAT status (unfilled = CAT-; blue fill = CAT+). All comparisons $p<0.05$ except for age, Temp, RR, chest bleeding, K+, and BUN. BDef, base deficit; Bld, bleeding; BUN, blood urea nitrogen; Cr, creatinine; DBP, diastolic blood pressure; FAST, Focused Assessment with Sonography in Trauma; GCS, Glasgow Coma Scale; Gluc, glucose; Hgb, hemoglobin; HR, heart rate; IVF, pre-hospital crystalloid; Lact, lactate; PTT, partial thromboplastin time; RR, respiratory rate; SBP, systolic blood pressure; Temp, temperature

(A)

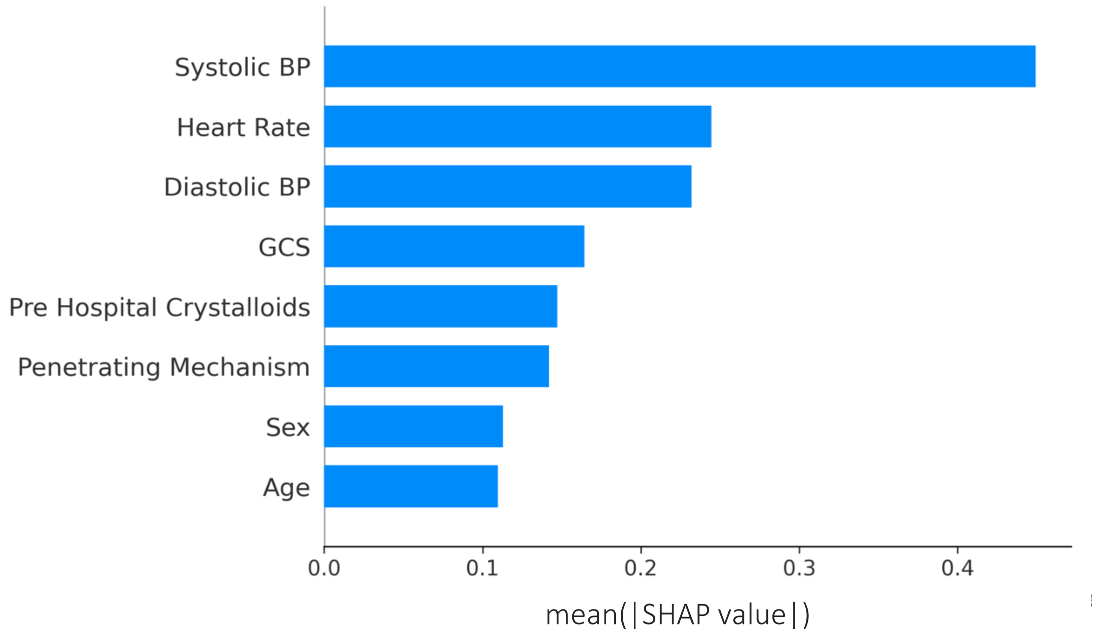

(B)

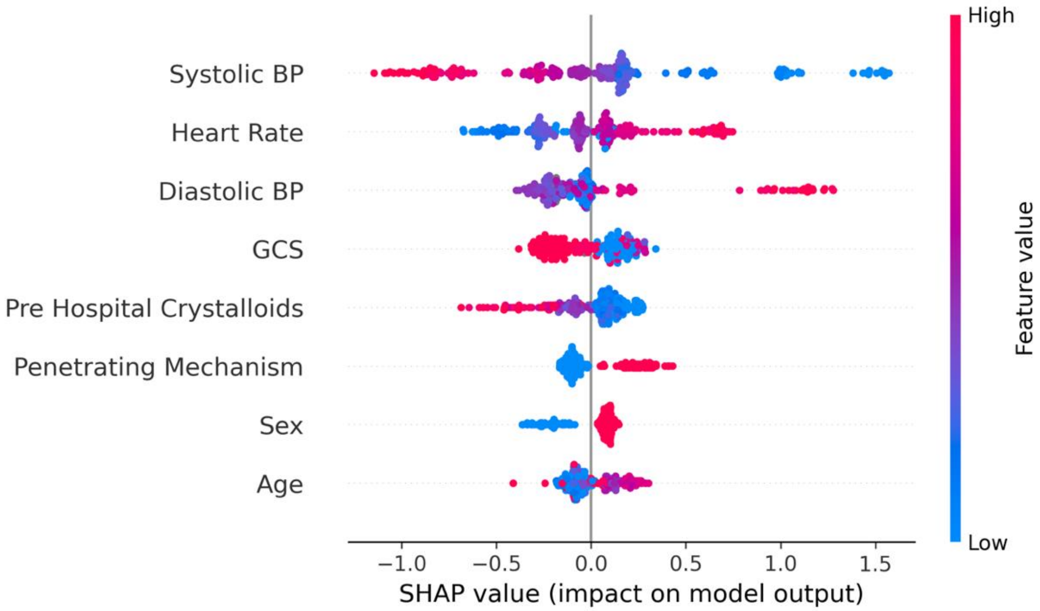

Supplemental Figure 2. Shapley additive explanation (SHAP) feature importance for Tier 1 variables. (A) Contribution of each variable to the model's decision-making ranked by mean of the absolute SHAP value as a measure of the average impact on model output magnitude. (B) Beeswarm plots demonstrating the association of each variable with the prediction of a CAT+ event, as identified by the model.

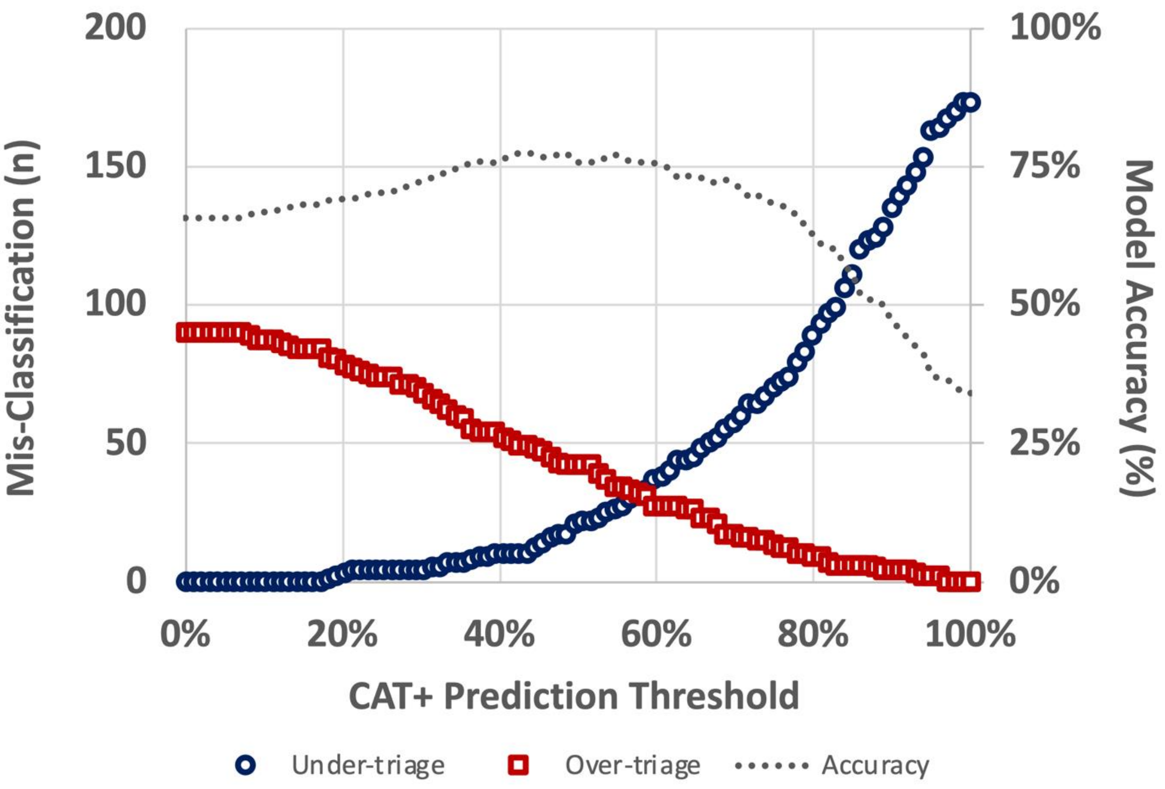

Supplemental Figure 3. Model performance in identifying CAT+ patients among a randomly selected 20% hold-out test set including both CAT+ (n=173) and CAT- (n=90) patients. At 42% CAT+ probability, the model successfully identified n=163 of 173 CAT+ patients for a false negative (under-triage) rate of 5.8%.

Supplemental Data: Blood Navigator Machine Learning Model Parameters

CAT+

Tier1: {'rsm': 0.06452933211, 'bootstrap_type': 'Bernoulli', 'od_wait': 50, 'boosting_type': 'Plain', 'od_type': 'Iter', 'iterations': 4753, 'loss_function': 'Logloss', 'l2_leaf_reg': 45, 'subsample': 0.7482778022, 'depth': 3, 'min_data_in_leaf': 17, 'learning_rate': 0.134266685}

Tier2: {'rsm': 0.0960288266, 'bootstrap_type': 'Bayesian', 'od_wait': 50, 'boosting_type': 'Ordered', 'od_type': 'Iter', 'iterations': 2911, 'bagging_temperature': 0.3118744205, 'loss_function': 'Logloss', 'l2_leaf_reg': 36, 'depth': 4, 'min_data_in_leaf': 276, 'learning_rate': 0.1836162962}

Tier3: {'rsm': 0.07093677373, 'bootstrap_type': 'MVS', 'od_wait': 50, 'boosting_type': 'Plain', 'od_type': 'Iter', 'iterations': 3053, 'l2_leaf_reg': 41, 'loss_function': 'Logloss', 'depth': 6, 'min_data_in_leaf': 266, 'learning_rate': 0.1997074026}

Tier4: {'rsm': 0.08708056548, 'bootstrap_type': 'MVS', 'od_wait': 50, 'boosting_type': 'Ordered', 'od_type': 'Iter', 'iterations': 618, 'l2_leaf_reg': 56, 'loss_function': 'CrossEntropy', 'depth': 12, 'min_data_in_leaf': 264, 'learning_rate': 0.1998788603}

Multiple CAT+:

Tier1: {'rsm': 0.08462104709, 'bootstrap_type': 'MVS', 'od_wait': 50, 'boosting_type': 'Plain', 'od_type': 'Iter', 'iterations': 4248, 'l2_leaf_reg': 44, 'loss_function': 'Logloss', 'depth': 9, 'min_data_in_leaf': 225, 'learning_rate': 0.1981321973}

Tier2: {'rsm': 0.08851621462, 'bootstrap_type': 'MVS', 'od_wait': 50, 'boosting_type': 'Plain', 'od_type': 'Iter', 'iterations': 1917, 'l2_leaf_reg': 17, 'loss_function': 'CrossEntropy', 'depth': 11, 'min_data_in_leaf': 168, 'learning_rate': 0.1794321301}

Tier3: {'rsm': 0.07783884667, 'bootstrap_type': 'MVS', 'od_wait': 50, 'boosting_type': 'Ordered', 'od_type': 'Iter', 'iterations': 744, 'l2_leaf_reg': 2, 'loss_function': 'CrossEntropy', 'depth': 10, 'min_data_in_leaf': 245, 'learning_rate': 0.1746396847}

Tier4: {'rsm': 0.02556779483, 'bootstrap_type': 'MVS', 'od_wait': 50, 'boosting_type': 'Plain', 'od_type': 'Iter', 'iterations': 3969, 'l2_leaf_reg': 4, 'loss_function': 'Logloss', 'depth': 3, 'min_data_in_leaf': 112, 'learning_rate': 0.1992942657}

Massive Transfusion

Tier1: {'rsm': 0.09596324952, 'bootstrap_type': 'Bernoulli', 'od_wait': 50, 'boosting_type': 'Plain', 'od_type': 'Iter', 'iterations': 481, 'loss_function': 'CrossEntropy', 'l2_leaf_reg': 25, 'subsample': 0.4015479387, 'depth': 3, 'min_data_in_leaf': 186, 'learning_rate': 0.1996577555}

Tier2: {'rsm': 0.09644278908, 'bootstrap_type': 'MVS', 'od_wait': 50, 'boosting_type': 'Plain', 'od_type': 'Iter', 'iterations': 1434, 'l2_leaf_reg': 3, 'loss_function': 'CrossEntropy', 'depth': 6, 'min_data_in_leaf': 280, 'learning_rate': 0.1840648883}

Tier3: {'rsm': 0.09272314891, 'bootstrap_type': 'Bayesian', 'od_wait': 50, 'boosting_type': 'Plain', 'od_type': 'Iter', 'iterations': 3927, 'bagging_temperature': 5.025838079, 'loss_function': 'CrossEntropy', 'l2_leaf_reg': 14, 'depth': 6, 'min_data_in_leaf': 75, 'learning_rate': 0.1828527528}

Tier4: {'rsm': 0.09982656319, 'bootstrap_type': 'Bayesian', 'od_wait': 50, 'boosting_type': 'Plain', 'od_type': 'Iter', 'iterations': 2003, 'bagging_temperature': 6.729475665, 'loss_function': 'CrossEntropy', 'l2_leaf_reg': 20, 'depth': 3, 'min_data_in_leaf': 123, 'learning_rate': 0.1995382789}

Mortality

Tier1: {'rsm': 0.08828734704, 'bootstrap_type': 'Bernoulli', 'od_wait': 50, 'boosting_type': 'Ordered', 'od_type': 'Iter', 'iterations': 683, 'loss_function': 'Logloss', 'l2_leaf_reg': 0, 'subsample': 0.1671936178, 'depth': 6, 'min_data_in_leaf': 101, 'learning_rate': 0.1544583688}

Tier2: {'rsm': 0.05428998515, 'bootstrap_type': 'Bernoulli', 'od_wait': 50, 'boosting_type': 'Ordered', 'od_type': 'Iter', 'iterations': 3077, 'loss_function': 'CrossEntropy', 'l2_leaf_reg': 100, 'subsample': 0.1004651452, 'depth': 2, 'min_data_in_leaf': 79, 'learning_rate': 0.1483976203}

Tier3: {'rsm': 0.09313057728, 'bootstrap_type': 'MVS', 'od_wait': 50, 'boosting_type': 'Ordered', 'od_type': 'Iter', 'iterations': 4484, 'l2_leaf_reg': 0, 'loss_function': 'CrossEntropy', 'depth': 2, 'min_data_in_leaf': 220, 'learning_rate': 0.1807911034}

Tier4: {'rsm': 0.09217591799, 'bootstrap_type': 'MVS', 'od_wait': 50, 'boosting_type': 'Ordered', 'od_type': 'Iter', 'iterations': 2001, 'l2_leaf_reg': 2, 'loss_function': 'Logloss', 'depth': 3, 'min_data_in_leaf': 27, 'learning_rate': 0.1650881697}
